# ﻿Mating and egg-laying behaviour of the Southeast Asian apple snail *Pilapesmei* (Morlet, 1889) (Caenogastropoda, Ampullariidae)

**DOI:** 10.3897/zookeys.1231.138263

**Published:** 2025-03-10

**Authors:** Supunya Annate, Ting Hui Ng, Chirasak Sutcharit, Somsak Panha

**Affiliations:** 1 Animal Systematics Research Unit, Department of Biology, Faculty of Science, Chulalongkorn University, 254 Phayathai Road, Pathumwan, Bangkok 10330, Thailand Chulalongkorn University Bangkok Thailand; 2 Institute for Tropical Biology and Conservation, Universiti Malaysia Sabah, Jalan UMS, 88450 Kota Kinabalu, Sabah, Malaysia Universiti Malaysia Sabah Kota Kinabalu Malaysia; 3 Academy of Science, The Royal Society of Thailand, Bangkok 10300, Thailand Academy of Science, The Royal Society of Thailand Bangkok Thailand

**Keywords:** Copulation, female resistance, freshwater snail, oviposition, reproduction, reproductive behaviour, spawning, video recording

## Abstract

The apple snail *Pilapesmei* is an economically valuable freshwater snail in mainland Southeast Asia whose populations have recently dramatically declined. Although conservation concerns have been rising in the region, the lack of basic knowledge of its reproductive biology remains an obstacle to its conservation. This study describes the mating and egg-laying behaviours of *P.pesmei* under laboratory conditions using continuous video recordings. Fifteen mating behaviours were recorded in four mating phases: pre-courtship, courtship, copulation, and post-copulation. Mating sequences were variable, especially in the courtship phase. However, many males performed a common courtship sequence of mate probing, mounting, shell circling, and positioning. When a female performed a variety of actions against the male’s approach, males then performed various alternative courtship sequences. The egg-laying process was similar among different females. They burrowed into the soil substrate to lay eggs. A total of six behaviours were recorded in the egg-laying process: crawling, resting, withdrawal, burrowing, egg depositing, and aestivating. Females did not return to the water after laying eggs but remained in the egg-laying cavity with their egg masses. This behaviour has not been reported in any other *Pila* species to date. Overall, our examination revealed the previously unknown mating and egg-laying processes in *P.pesmei*, which included some distinct behaviours and increased the basic knowledge of its reproductive biology.

## ﻿Introduction

The Southeast Asian apple snail, *Pilapesmei* (Morlet, 1889), is a freshwater snail belonging to the family Ampullariidae. *Pilapesmei* has been reported in all the mainland Southeast Asian countries: Cambodia, Laos, Myanmar, Thailand, and Vietnam (Keawjam 1986, 1990; Cowie 2015; Ng et al. 2020a, 2020b). It is one of the economically valuable freshwater snails in the region. Natural populations of *P.pesmei* have been exploited for food acquisition and trading (Ngor et al. 2018; Ng et al. 2020b). Recently, populations of *P.pesmei*, like other Southeast Asian apple snails have decreased due to several likely factors, including habitat degradation, overexploitation, and introduction of the South American apple snails *Pomaceacanaliculata* (Lamarck, 1822) and *Pomaceamaculata* Perry, 1810 (Ngor et al. 2018; Marwoto et al. 2020; Ng et al. 2020a). Although concern for the conservation of these species has been rising in the last few years, effective conservation action is still hard to enact because of the lack of knowledge regarding their reproductive biology (Annate et al. 2023).

There is no study of the reproductive biology of *P.pesmei* to date. A few details of its reproductive biology have been scattered in reports of taxonomic or ecological studies (Keawjam 1987; Lamkom and Phosri 2017; Ng et al. 2020a). *Pilapesmei* is dioicous. Male and female snails are indistinguishable by external morphology. Shell sizes of mature snails range from 30 to 60 mm long and from 27 to 50 mm wide (Keawjam 1986; Ng et al. 2020a). However, the mating process in this species is unknown and remains to be described.

The egg-laying behaviour of *P.pesmei* has been briefly mentioned by Lamkom and Phosri (2017) and Ng et al. (2020a), but these two studies reported slightly different egg-laying behaviours. Based on casual observation, the species was reported to lay approximately 100 white spherical eggs in a shallow cavity away from water in the wild (Ng et al. 2020a), whereas the ex-situ study (Lamkom and Phosri 2017) reported that it lays 107–301 white eggs on the soil.

Because of these scattered and confusing details, it is necessary to investigate the reproductive behaviour of *P.pesmei*. Building on previous research documenting the behaviours of *Pila* species (Annate et al. 2023), herein we report the mating and egg-laying behaviours of *P.pesmei*.

## ﻿Methods

### ﻿Materials

We collected 70 mature snails (shell length over 30 mm) from a population inhabiting a paddy field in Mukdahan province, Thailand (16°42'46"N, 104°42'36"E) on 25 May 2023 when they had first emerged from aestivation following the first flood after the dry season. Snails were transported to the laboratory on the same day and sexed according to the presence of the penis sheath. From the 33 males and 37 females collected, eight males and eight females were used in the first egg-laying behaviour trial (video recorded). The other 25 males and 29 females were maintained separately in 0.45 m^2^ circular concrete containers, each containing 80 l of tap water, at a maximum density of 10 snails/container for 30 days. They were then used in mating behaviour trials (video recorded) and the second and third egg-laying behaviour trials (also video recorded). Snails were fed with lettuce *ad libitum*. Water was changed and maintenance containers were cleaned monthly.

### ﻿Mating behaviour: (i) video recording

Prior to video recording the mating behaviour, snails were transferred from the maintenance containers to a 17.5-l glass aquarium containing 10 l of tap water for acclimatisation. Males and females were kept in separate aquaria at a stocking density of two snails/aquarium at ambient temperature (29 ± 1 °C) under a 12:12 h photoperiod. They were acclimatised for 24 h and were fed with lettuce *ad libitum* during this period.

To video record the mating behaviour, one male and one female were randomly transferred to a 17.5-l glass aquarium containing 10 l of tap water. Five pairs were recorded concurrently in each of a total of 10 trials in July and August 2023. Snail behaviour was recorded for 24 h from when they were paired using an infrared digital video camera (5 MP HD IP Camera, Model YM500L) installed in front of the aquarium (one per aquarium).

After video recording, females that had mated were transferred to an egg-laying aquarium where they were kept to screen for oviposition. Males that had mated were transferred to new maintaining containers. Snails that had not mated were returned to their maintaining containers and reused for subsequent mating behaviour video recording trials after 24 h.

### ﻿Mating behaviour: analysis of video recordings

**Figure 1.**
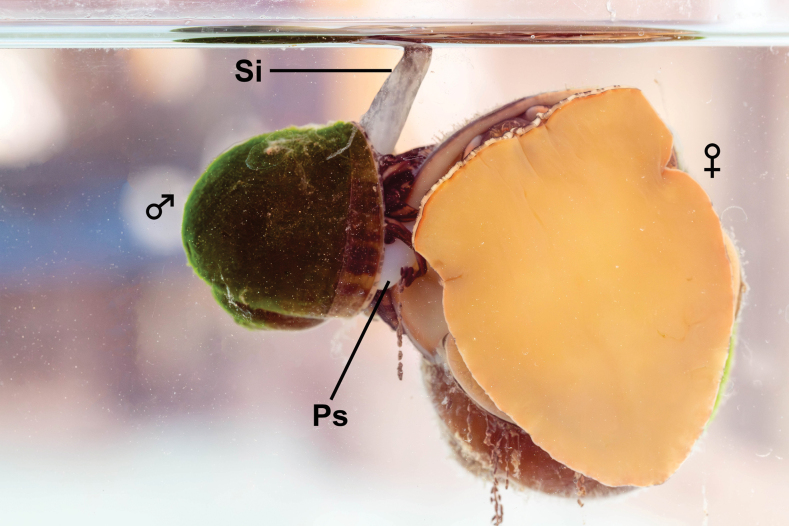
Insemination posture of *Pilapesmei*. Abbreviations: Ps, penis sheath; Si, siphon.

Video records of the mating behaviour of *P.pesmei* were first thoroughly scanned for the presence of an insemination posture (Fig. 1). The video record of each pair was then analysed by watching at 1–8× of the normal speed rate. Behaviour of each mating pair was analysed from the beginning of the video record, when the pairs were released into the mating aquaria, until one hour after they had dismounted from each other. Behaviour of non-mating pairs was analysed for 4 h from their initial release into the mating aquarium.

Mating behaviours of *P.pesmei* were classified based on the descriptions for *P.virescens* (Deshayes, 1824) (Annate et al. 2023) and *Pomaceacanaliculata* (Burela and Martín 2009). The mating process was divided into the four phases, following Burela and Martín (2009): 1. Pre-courtship phase, the period from the beginning to the first contact between two snails; 2. Courtship phase, the period from the first contact between two snails to the time point that the male inserts the penis sheath into the female genital pore; 3. Copulation phase, the period from penis insertion to penis withdrawal; and 4. Post-copulation, the period from penis withdrawal to the time point that the two snails separated from each other.

All the observed behaviours that may be related to mating were then described with a diagram of the mating sequences. The duration of the entire mating process and each of the behavioural phases were recorded in hours and presented as the mean ± one standard deviation (SD).

### ﻿Egg-laying behaviour: (i) video recording

Each egg-laying arena was a 0.45 m^2^ circular concrete container housing the water body (40 l of tap water) and the soil area for oviposition. Snail behaviour was recorded for 30 days using one infrared digital video camera installed 1.5 m above each of the two arenas in each of three trials.

Four males and four females were randomly selected and transferred from the maintenance concrete containers to an egg-laying arena. Before releasing the snails into the egg-laying arenas, the shell width and shell length were measured using digital Vernier calipers to the nearest millimeter. Snails were marked using nail polish for sex identification in the subsequent video examination. The first trial was conducted between 25 May and 25 June 2023, the second between 28 June and 28 July 2023, and the third between 30 July and 30 Aug 2023. At the end of each trial, snails were removed from the arenas. The soil areas were searched for egg masses. Each arena was cleaned before conducting the next trial.

### ﻿Egg-laying behaviour: (ii) video examination

Video records were scanned for the presence of females in the soil areas away from the water body or for any burrowing behaviour every 10 min. When the females showed a burrowing behaviour, we re-examined the video records by continuously watching at 1–8× of the normal speed from the time point that the female started crawling out of the water until the female finished egg laying or returned to the water.

All the observed behaviours in the egg-laying process were then described with a diagram of the egg-laying sequences. The duration of each behaviour in the egg-laying process was recorded in hours and presented as the mean ± SD.

## ﻿Results

### ﻿Mating behaviour

Among the 50 recorded pairs, six pairs mated, and the other 44 pairs did not mate. Among the 44 non-mating pairs, eight did not show any visible behavioural interaction in the first 4 h, while the other 36 non-mating pairs showed some behavioural interactions. Therefore, a total of 42 pairs (six mating pairs and 36 non-mating pairs) were included in the mating behaviour analysis.

**Figure 2.**
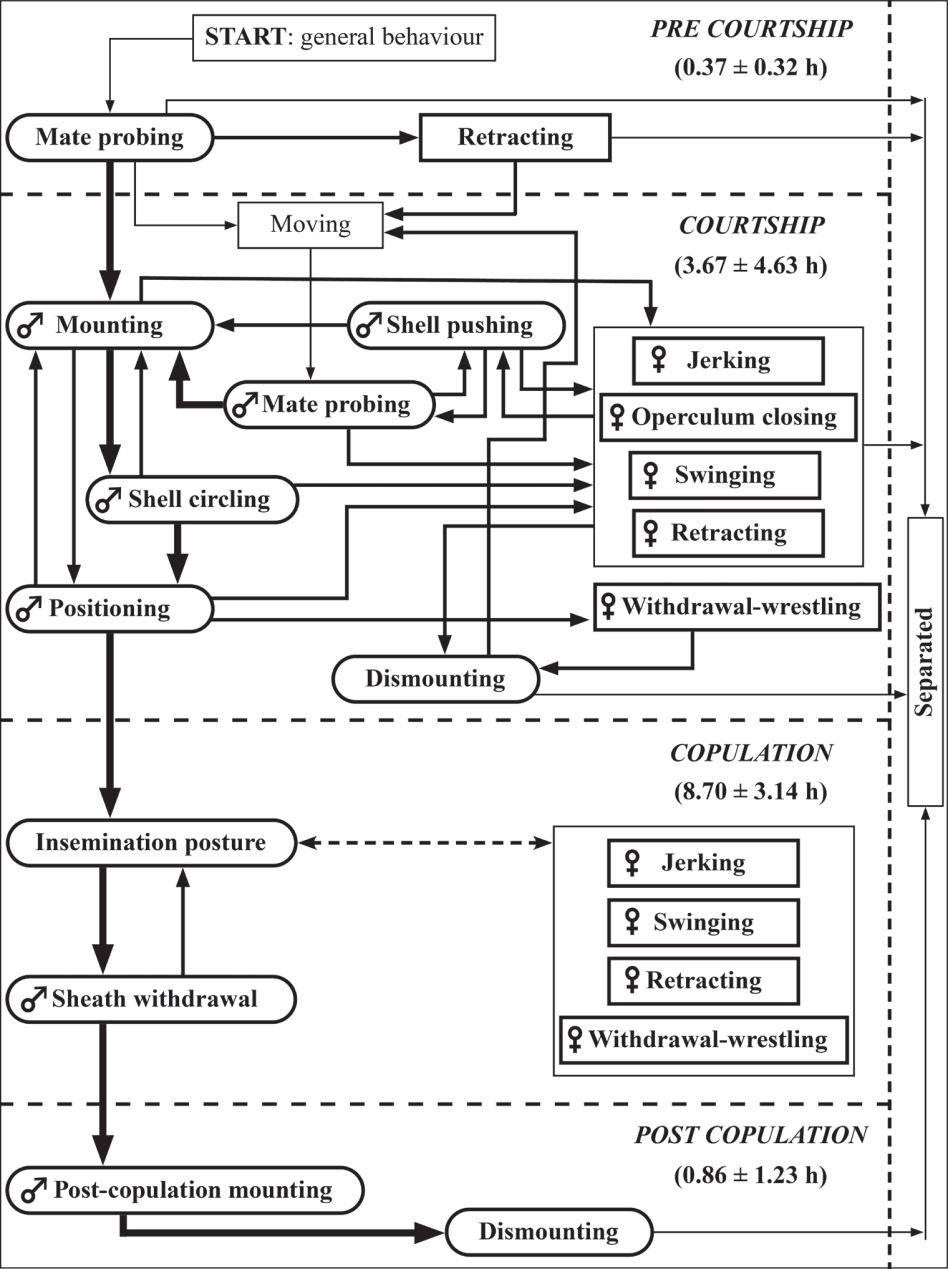
Mating sequences of *Pilapesmei*. Thick-rounded boxes represent mating behaviour. Thick-straight boxes represent female resistance behaviour. Solid arrows indicate behavioural transitions of the main mating sequences. Moderate arrows indicate behavioural transitions of the alternative mating behavioural sequences. Thin arrows indicate behavioural transitions between general and mating behaviours. The hatched arrow indicates the co-occurrence of the insemination posture and female resistance behaviour. Durations of each phase are provided in brackets (mean ± SD).

Paired snails showed 15 distinguishable behaviours in the mating process. Names and descriptions of each behaviour are provided in Table 1. The mating process included four phases within which the behavioural interactions and sequences were variable (Fig. 2). The pre-courtship phase included breathing, resting, withdrawal, and walking behaviours. When each pair was released into the aquarium, they withdrew their body into the shell and the operculum completely closed the aperture. Then, they extruded their tentacles and cephalopodium while opening the operculum slightly. The first contact between the male and female occurred while they moved around in the aquarium. In 29 out of the 42 pairs, males contacted females first, whereas females contacted males first in the other 13 pairs. After the first contact, four pairs showed no further behavioural interaction in the 4 h; thus, these pairs did not enter the courtship phase. The other 38 pairs entered the courtship phase performing various behavioural interactions.

**Table 1.** Descriptions of the mating behaviour of *Pilapesmei* (applied from Annate et al. 2023).

Behavioural type	Behaviour	Description
**Mating behaviour**		
	Mate probing	Contact with cephalic tentacles, labial palps, or foot.
	Shell pushing	Push another snail by moving its shell towards another snail, generally performed by males in response to a female resistance behaviour especially retracting.
	Mounting	Mount on another snail with the foot having completely lost its hold on the aquarium.
	Shell circling	Crawl on another snail’s shell and move over it in either a clockwise or counterclockwise direction.
	Positioning	Male adheres on the female’s last whorl at the right side of the shell rim above the opening of the female gonopore.
	Insemination posture	Male adheres to the right side of the female’s last whorl with coiled tentacles. Female usually motionless, but occasionally moves around.
	Sheath withdrawal	Male withdraws penis sheath from female gonopore.
	Post-copulation mounting	Male continues mounting on the female shell after sheath withdrawal.
	Dismounting	Male detaches from the female’s shell.
	Passive	Motionless, while copulation with coiled tentacles; male tightly adheres to the female’s shell, female tightly adheres to or detaches from the aquarium.
**Resistance behaviour**		
	Jerking	Contraction and release cephalopodium into- and out of the shell several times.
	Operculum closing	Quickly close the aperture with their operculum in response to any contacts from another snail.
	Retracting	Contraction of cephalopodium into the shell and tightly adhere to or completely detach from the aquarium.
	Swinging	Rotate the shell several times in a counter- and clockwise directions.
	Withdrawal-wrestling	Female pushes the male’s shell with her operculum at the rim of the male aperture.

In 16 of the 38 pairs, the male immediately courted the female by pushing, mounting, and circling on the female’s shell following the first contact. In contrast, the other 22 pairs did not perform any immediate subsequent courtship behaviour following the first contact. Rather, they began courting after later contacts. Among the 38 pairs that entered the courtship phase, 17 pairs performed only one courtship behaviour, which was mate probing. The other 21 pairs performed variable sequences of courtship behaviours, such as mate probing and shell pushing or mate probing and mounting (Fig. 2). However, the courtship sequences that led to copulation were almost the same. A total of six pairs that courted then entered the copulation phase. Five of these six pairs performed the same courtship sequence of four behaviours: mate probing, mounting, shell circling, and positioning. For the other pair, the male did not perform shell circling but mounted on the female shell near the aperture above the gonopore.

In 14 pairs, the female did not show a distinctive behaviour after being approached or courted by a male. In the other 24 pairs, the female performed five types of resistance behaviours: jerking, operculum closing, retracting, swinging, and withdrawal-wrestling (see description in Table 1).

The copulation phase began after the male positioned himself on the right side of the rim of the female’s last whorl above her gonopore. The male then inserted a penis sheath into the female pallial cavity, and the pair stayed still. The process of penis sheath insertion was not recorded in this study because all penis sheath insertion happened when mating pairs were on the side of the aquarium with no camera facing. However, the beginning of the copulation phase was judged based on the combination of male and female behaviours: the male had a coiled tentacle and tightly adhered to the rim of the female’s last whorl above the gonopore, while the female suddenly ceased all moving and withdrew the soft body staying on the aquarium wall. After that, the mating pairs remained in the insemination posture (Fig. 1) for several hours. During this period, the mating pairs were generally in a passive state, staying still in the same position. Sometimes, copulating females crawled around, mainly to the water surface for gas exchange. In addition, five of the six copulating females performed some resistance behaviours simultaneously with being passive or crawling. Four resistance behaviours: jerking, retracting, swinging, and withdrawal-wrestling were recorded in this phase. The male’s posture was consistent since the beginning of copulation. Males moved only their siphons for gas exchange when the mating pairs were near the water surface. When the copulating females crawled on the aquarium wall in front of the camera, the male’s penis sheath was seen clearly (Fig. 1).

At the end of the copulation phase, males withdrew the penis sheath from the female pallial cavity. At the same time, males started uncoiling their tentacles and moving. In response, females retracted their soft bodies strongly but did not close the operculum. After copulation, five of the six mating pairs entered the post-copulation phase. The other pair copulated again and then entered the post-copulation phase.

In the post-copulation phase, the male stayed on the female’s shell after penial separation from the female’s pallial cavity. They either adhered to the female’s shell without movement or crawled over the female’s shell for several rounds. At the end of the post-copulation phase, the male dismounted from the female’s shell, and moved to the water surface for gas exchange, after which they remained adhered to the aquarium wall, entering the resting stage for over one hour with only a few slight movements. In contrast, females moved around in the mating aquaria and often climbed out of the water on the aquarium wall.

### ﻿Egg-laying behaviour

**Figure 3.**
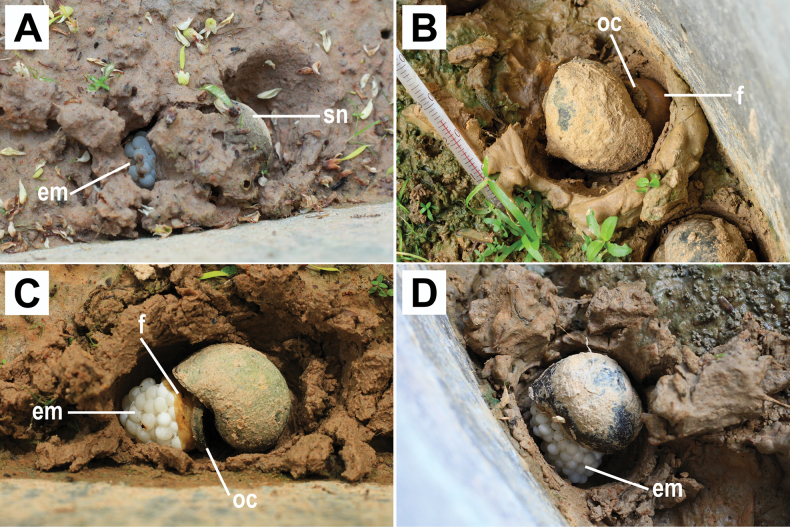
Egg-laying behaviour of *Pilapesmei*. **A** female lays eggs in an egg-laying cavity that is obscured by soil **B** female’s foot becomes a dome shape **C** egg mass exceeds the foot coverage and can be seen clearly **D** female stays on top of the egg mass after finishing egg laying. Abbreviations: em, egg mass; f, foot; oc, operculum; sn, snail.

A total of 11 females laid eggs in the egg-laying observation containers. They burrowed into the soil and laid eggs within the burrow. Five females burrowed deep into the soil and remained out of view (Fig. 3A). Consequently, six egg-laying occasions were recorded and examined in this study. In the three phases of the egg-laying process, females performed six different behaviours (Table 2).

**Table 2.** Descriptions of the egg-laying behaviour of *Pilapesmei*.

Stage	Behaviour	Description
**Pre-egg depositing**	Crawling	Crawling out of water onto land and moving around on land.
	Resting	Stop crawling or digging and stay motionless while still extruding the soft body out of the shell.
	Withdrawal	Complete withdrawal of the soft parts into the shell and closing of the aperture with the operculum.
	Burrowing	Digging into the soil to make an egg-laying cavity.
**Egg depositing**	Egg depositing	Laying eggs to form an egg mass within an egg-laying cavity.
**Post-egg depositing**	Aestivating	Complete closing of the aperture with the operculum and staying inside the egg-laying cavity.

**Figure 4.**
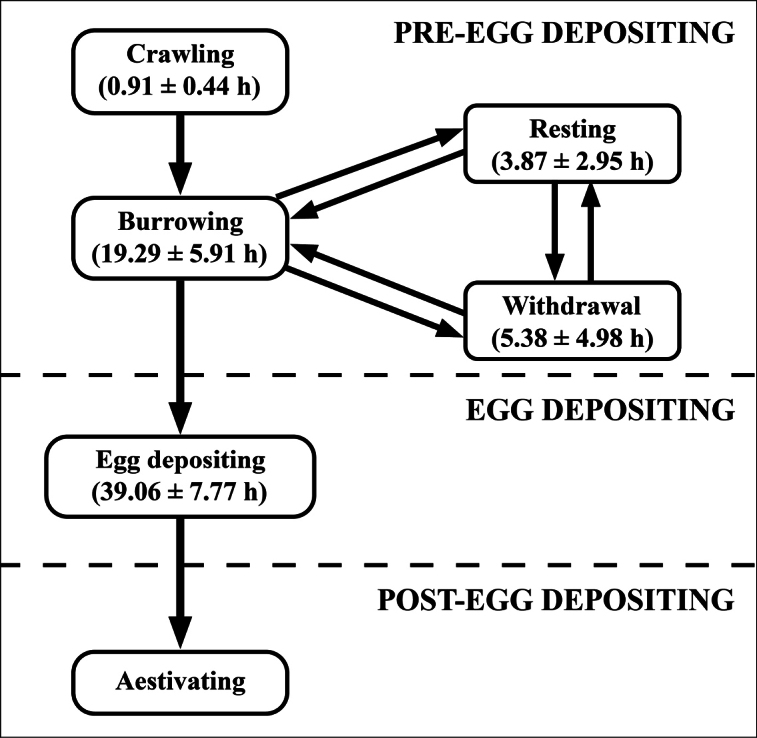
Egg-laying sequences of *Pilapesmei.* Thick-rounded boxes represent each egg-laying behaviour with the duration of the behaviour provided in brackets (mean ± SD). Arrows indicate behavioural transitions of the egg-laying sequences.

Egg-laying sequences were simple and similar among the females (Fig. 4). Females crawled out of the water body in the nighttime onto the soil area and then moved around on the soil surface. After that, females burrowed into the soil making a cavity. While making a cavity, they sometimes stopped burrowing and stayed motionless while extruding their soft body or withdrawing it inside the shell and closing the aperture with an operculum. Females took 10–24 h to make the egg-laying cavity.

The depth of the egg-laying cavity ranged from 3 to 5 cm. After the egg-laying cavity was completed, females expanded their feet roundly. After a few hours, the female’s foot became dome-shaped (Fig. 3B), under which eggs were sometimes visible through a thin area of the female’s foot. After several hours, the egg mass exceeded the foot coverage (Fig. 3C). At the end of egg laying, females closed the aperture with the operculum and stayed in the egg-laying cavity beside or on top of their egg masses (Fig. 3D). Until the end of the video recording period, they did not return to the water in the containers. When transferred into the water, they began to move around.

## ﻿Discussion

This study provides the first description of the mating and egg-laying behaviours of *P.pesmei* under laboratory conditions. Male and female pairs performed 15 different behaviours in four mating phases. Females performed six egg-laying behaviours in three egg-laying phases.

In general, the mating process of *P.pesmei* is similar to the common mating pattern of other apple snails: male mounts on the female’s shell, crawls on the female’s shell, positions himself on the right side of the female’s last whorl above her gonopore, inserts the penis sheath into the female’s pallial cavity, and mates for several hours (Albrecht et al. 1996; Burela and Martín 2007, 2009; Tiecher et al. 2014; Gurovich et al. 2017; Annate et al. 2023). However, the behavioural interactions between males and females and the behavioural transitions between different behaviours in the courtship phase of *P.pesmei* are variable (Fig. 2). Courtship variations mainly occurred in the mating pairs in which the females performed resistance behaviour. A clear example of consistent courtship variation is the performing of shell pushing. In courtship bouts without female resistance, the male performed mate probing and mounting. In some of the courtship bouts with female resistance, the male sequentially performed mate probing, shell pushing, and mounting. Such variation is possibly an alternative male reproductive tactic that is influenced by female mate choice (Oliveira et al. 2008).

Female resistance has previously been reported in two apple snails: *Pomaceacanaliculata* (Burela and Martín 2009) and *P.virescens* (Annate et al. 2023). Burela and Martín (2009) reported three types of female resistance: operculum closing, swinging, and withdrawal-wrestling and suggested these behaviours are mate-rejection actions because these behaviours can cease mating. In our previous study on the mating behaviour of *P.virescens* (Annate et al. 2023), two more female resistance behaviours that can cease the mating process of *P.virescens* were identified: jerking and retracting. In addition, these five female resistance behaviours have been suggested as mate-rejection behaviours in several freshwater snails (Barraud 1957; DeWitt 1991; Facon et al. 2006; Tiecher et al. 2014). In *P.pesmei*, five types of female resistance behaviour, which have previously been reported in other apple snails, were recorded. Female resistance can cease some courtship bouts of *P.pesmei*. Therefore, it is possibly a mate-rejection behaviour as suggested in previous studies on other snail species (Burela and Martín 2009; Annate et al. 2023).

Nevertheless, it is unknown why several females rejected their given partner in this study. Immaturity is unlikely to be the reason because some of these females mated and laid eggs when reused in the egg-laying behaviour video recording. Two possible reasons for females to avoid copulation are (1) they are ready to lay eggs and (2) they prefer to mate with a suitable male. Snails used in this study were collected from nature, and so they may have mated before being collected. Although they were maintained separately for 30 days before use in the video recording, it is possible that females can store sperm for over 30 days. Sperm-storing ability of over 30 days has been reported in some apple snails (Albrecht et al. 1996; Estebenet and Martín 2002; Tiecher et al. 2014). If female *P.pesmei* can store sperm, they may then avoid excessive mating with the given experimental male.

Female mate choice has never been reported in apple snails but cannot be ruled out because some females that did not mate in the video recorded mating behaviour trials mated when they were kept in the collective container where several males were present. Presumably, they rejected the given partner but accepted the suitable mate in the collective container.

Should female mate choice exist in this species, it is possible that female resistance is also related to their assessment of the male because five of the six copulating females performed a clear resistance behaviour in the courtship and copulation phases but finally entered the passive stage of the insemination posture. These females may perform resistance behaviour to screen for a suitable mate, for example, rotating their shells to evaluate male size and weight. One potential evidence for this assumption is that four of the six copulating females performed three types of resistance behaviour—jerking, retracting, and swinging—that resulted in shell rotation or movement in different directions. Although these behaviours were performed several times, none of them interrupted the copulation.

There was one mating pair in which the copulation was interrupted. The most noticeable difference in the mating process of this pair compared to the other mating pairs was the withdrawal-wrestling behaviour. The female performed withdrawal-wrestling in the early copulation phase. Although the pair entered the passive stage of the insemination posture at first, the male withdrew the penis sheath from the female pallial cavity after being in the passive stage for 2 h and then reinserted the penis sheath and entered the insemination posture again. Penis sheath withdrawal in the early stage of the insemination posture has previously been reported in *Pomaceacanaliculata*. It was assumed that the male withdrew the penis sheath because it could not find the female gonopore or the female closed the gonopore as a last mate-rejection mechanism (Burela and Martín 2009). Following these assumptions, withdrawal-wrestling may play a part in disturbing the penis sheath intromission of *P.pesmei* and, as such, it would be a clear mate-rejection behaviour.

At the end of the mating process, the male dismounted from the female’s shell, and the mating pair separated (Burela and Martín 2009; Annate et al. 2023). We followed the behaviour of the snails for one hour after dismounting. All the males spent most of the time resting, and none performed courtship again during this period. It is possible that they need to recover after the energy expenditure during mating. In contrast to males, females spent most of their time moving around in the water and frequently crawling above water. Presumably, they moved above water to find an area for laying eggs.

Apple snails in the genus *Pila* exhibit various aerial egg-laying behaviours, such as *P.globosa* (Swainson, 1822) lays eggs in a hollow on a pond bank (Bahl 1928), *P.virescens* lays eggs on soil near a water line or floating plants (Ng et al. 2020a; Annate et al. 2023), and *P.celebensis* (Quoy & Gaimard, 1834) lays eggs on hard vertical substrates above water (Djajasasmita 1987; Ng et al. 2020a). Most of the apple snails in the genus *Pomacea* lay eggs on hard vertical substrates above water (Hayes et al. 2009, 2015; Burks et al. 2010; Gurovich et al. 2017) except for one species, *Pomaceaurceus* (Müller, 1774), which burrows into the soil, then lays eggs in its shell, and aestivates with its eggs over the dry season (Burky et al. 1972). Previously, it was unclear whether *P.pesmei* lays eggs on the soil (Lamkom and Phosri 2017) or in a shallow cavity (Ng et al. 2020a). Here, our results agree with Ng et al. (2020a).

The egg-laying process of *P.pesmei* can be divided into three phases: pre-egg depositing, egg depositing, and post-egg depositing phases. The pre-egg depositing phase started at night when the females first crawled out of the water, which follows the general nighttime oviposition reported for other apple snails (Albrecht et al. 1996; Heiler et al. 2008; Gilal et al. 2015; Gurovich et al. 2017). However, since the egg-laying process of *P.pesmei* lasted over 24 h, then the full egg-laying process occurred during both the day and night.

Prior to laying eggs, *P.pesmei* burrows into the soil, at a depth of 3–5 cm to make an egg-laying cavity. The egg-laying cavity seems important for *P.pesmei* because the female took 10–24 h to make it. In addition, some females performed resting and withdrawal behaviours alternately with burrowing. This could represent the high energy expenditure of the female to burrow the cavity. Laying eggs in a cavity has been previously reported only in *P.globosa*, where the cavity was suggested to be a shelter to protect the eggs from predators, sunlight, and desiccation (Bahl 1928).

The egg-depositing process occurred in-between the ventral foot surface and the soil. Therefore, it was not recorded from the top view camera of the present experimental design. From the top view, the female posture was consistent between all the females and is similar to that reported for *P.globosa*: females protruded their feet roundly with their head parts moderately withdrawn and later their feet became dome shaped for holding and arranging the eggs into egg masses (Bahl 1928). Presumably, the egg-depositing process occurring in between the ventral foot surface and the soil is also similar between the two species.

After depositing eggs, most of the previously studied apple snail species returned to the water immediately or within a short period (Bahl 1928; Albrecht et al. 1996; Annate et al. 2023) except for one species, *Pomaceaurceus*, which incubates its eggs and aestivates over the dry season (Burky et al. 1972). In this study, *P.pesmei* did not return to the water, but then stayed in the egg-laying cavities with completely closed apertures. None of the females left their egg masses even after the eggs hatched 20–23 days later. Therefore, it is possible that females of *P.pesmei* may aestivate after laying eggs or that they protect and incubate their eggs, similar to that previously reported in *Pomaceaurceus*. However, oviposition in *Pomaceaurceus* mostly occurs at the beginning of the dry season and their hatchlings then aestivated until the next rainy season (Burky et al. 1972). In contrast, we found that the oviposition of *P.pesmei* was mainly observed during the first and second observation trials that were conducted between May and July, in the early part of the rainy season. In addition, some hatchlings of *P.pesmei* emerged from the egg-laying cavity and went into the water before the end of the video recording period. Thus, *P.pesmei* hatchlings may not need to aestivate immediately after hatching as they still have time over the remaining two months of the wet season to grow before the beginning of the dry season. However, for the females, it is questionable why females of *P.pesmei* did not return to the water after egg laying, since they laid eggs in the early rainy season and so do not need to immediately aestivate. We observed that some females in the collective containers, exposed to natural weather conditions, left their egg-laying cavity 7–10 days after laying the eggs following rain. Therefore, it can be assumed that they are more likely to stay in the egg-laying cavity to protect or incubate their eggs than to aestivate. Rain may be a key trigger inducing females to return to the water, explaining why females in the collective containers returned to the water when exposed to rain but those in the egg-laying observation containers, which were under roof cover, never returned to the water because they were never exposed to rain.

In conclusion, our examination into the details of the reproductive behaviour of the Southeast Asian apple snail, *P.pesmei*, revealed its previously unknown mating and egg-laying processes and confirmed the oviposition location of *P.pesmei* in a shallow cavity, as reported by Ng et al. (2020a). Since the oviposition location is a significant character for understanding apple snail evolutionary history (Hayes et al. 2009), confirming the oviposition location of *P.pesmei* contributes to a better understanding of the biology and evolution of the genus *Pila* and the family Ampullariidae. However, confirming the oviposition location of *P.pesmei* from different populations is still needed because the oviposition location of *P.pesmei* in the present study is contradictory to that which was previously reported by Lamkom and Phosri (2017). These contrary reports may reflect a variation in this species between different populations or habitats. Furthermore, Lamkom and Phosri (2017) identified their species as *P.ampullacea* (Linnaeus, 1758) instead, although the image provided, and the distribution and size reported indicated that the species is *P.pesmei*. Thus, it is also possible that the species studied by Lamkom and Phosri (2017) is a different species that shows a similar shell morphology to *P.pesmei*. Based on molecular data, Ng et al. (2020a) suggested the possibility of cryptic species within *P.pesmei* in Thailand. Owing to this uncertainty, we have not compared our data to the previous study by Lamkom and Phosri (2017). Rather, we recommend future research to explore the egg-laying behaviour of *P.pesmei* from different populations, combined with molecular species confirmation of each studied population, to broadly understand the behavioural variation patterns.

## ﻿Additional information

### Author ORCIDs

Supunya Annate https://orcid.org/0009-0001-6359-7954

Ting Hui Ng https://orcid.org/0000-0002-5123-0039

Chirasak Sutcharit https://orcid.org/0000-0001-7670-9540

Somsak Panha https://orcid.org/0000-0002-4431-2458

### Data availability

All of the data that support the findings of this study are available in the main text.
